# Supplementary material for: Identification of a conserved S2 epitope present on spike proteins from all highly pathogenic coronaviruses
Source: eLife. 2023 Mar 21;12:e83710. doi: 10.7554/eLife.83710 (PMC10030117; doi:10.7554/eLife.83710)
Supplement: Figure 1—source data 1. [file elife-83710-fig1-data1.zip › Spike HDX summary table.docx]

**Fig. 1 Source Data. HDX summary table**

| **Data Set** | **SARS-2 HexaPro spike** | **SARS-2** | **SARS-2** |
| --- | --- | --- | --- |
|  |  | **HexaPro spike** | **HexaPro spike** |
|  |  | **+ 3A3 IgG** | **+ 3A3 Fab** |
| **HDX reaction details** | 200 mM NaCl, 20 mM Tris | | |
|  | 0.5 μM S2 | 0.5 μM S2 | 0.5 μM S2 |
|  |  | 0.55 μM 3A3 IgG | 0.55 μM 3A3 Fab |
|  | pH_read_ = 7.6 | | |
| **HDX time course (s)** | 10, 100, 1000, 10000 at 25°C | | |
| **HDX control samples** | Unlabeled S2 | | |
| **Back-exchange (mean %)** | ~40 | | |
| **# of peptides** | 192 | | |
| **Sequence coverage (%)** | 56.3 | | |
| **Average peptide length (aa)/redundancy** | 13/3.34 | | |
| **Replicates (biological or technical)** | 4 (technical) | | |
| **Average standard deviation (Da)** | 0.10 | | |
| **Significance** | Average ΔHDX >0.2 Da, *p*-value<0.01 | | |
