# Supplementary material for: Identification of a conserved S2 epitope present on spike proteins from all highly pathogenic coronaviruses
Source: eLife. 2023 Mar 21;12:e83710. doi: 10.7554/eLife.83710 (PMC10030117; doi:10.7554/eLife.83710)
Supplement: Supplementary file 1. [file elife-83710-supp1.zip › 3A3 IgG HDX summary table.docx]

**Supplementary File 1a. HDX summary table**

| **Data Set** | **3A3 IgG** | **3A3 IgG**  **+ Spike** |
| --- | --- | --- |
| **HDX reaction details** | 200 mM NaCl, 20 mM Tris | |
|  | 0.5 μM 3A3 IgG | 0.5 μM 3A3  0.75 μM S2 |
|  | pH_read_ = 7.6 | |
| **HDX time course (s)** | 10, 100, 1000 at 25°C | |
| **HDX control samples** | Unlabeled 3A3 IgG | |
| **Back-exchange (mean %)** | ~40 | |
| **# of peptides** | 87 (heavy chain), 82 (light chain) | |
| **Sequence coverage (%)** | 73.8 (heavy chain), 87 (light chain) | |
| **Average peptide length (aa)/redundancy** | 13/3.21 (heavy chain), 4.84 (light chain) | |
| **Replicates (biological or technical)** | 4 (technical) | |
| **Average standard deviation (Da)** | 0.08 | |
| **Significance** | Average ΔHDX >0.15 Da, *p*-value<0.01 | |
