# Supplementary material for: Identification of a conserved S2 epitope present on spike proteins from all highly pathogenic coronaviruses
Source: eLife. 2023 Mar 21;12:e83710. doi: 10.7554/eLife.83710 (PMC10030117; doi:10.7554/eLife.83710)
Supplement: Supplementary file 3. — Peptides were selected from regions identified as having bimodal distributions in Costello et al. We had no coverage in residues 626–636 and 1146–1166. Peak width (PW) in Da was calculated in triplicate for each peptide in the non-deuterated sample (ND Control) and at each time point of exchange. The SD is reported. The change in peak width (ΔPW) was calculated by subtracting the PW of the control from the PW of the sample. Bimodality was assessed by taking the maximum peak width for a particular peptide (ΔPWmax) and assessing if it was greater than 2 Da. Peak width was calculated using a method similar to Weis et al., 2006. Peptides were centroided with the Apex3D algorithm using DynamX (Waters). Following manual curation, ion stick data were transferred into Excel as two columns of data, m/z values and intensities, and the maximum peak in the isotopic envelope was determined. The list was then searched in descending m/z order to identify the two lowest m/z peaks that straddled 20% of the maximum peak intensity. The m/z value at an envelope intensity of 20% of the maximum intensity was determined using linear interpolation between these two peaks. This process was repeated with a search in ascending m/z order. The relative peak width was determined by multiplying by z (the charge state). For peptide spectra without peaks straddling 20% of the maximum peak intensity on one side of the maximum, typical for lower m/z peaks for peptides exhibiting low deuteration, the farthest isotopic centroid peak on that side was used as the m/z limit for calculating peak width while the other m/z limit was determined using the previously described method. [file elife-83710-supp3.docx]

**Supplementary File 1c. Width of Isotopic Distributions for Example Spike Peptides.** Peptides were selected from regions identified as having bimodal distributions in Costello et al. We had no coverage in residues 626-636 and 1146-1166. Peak width (PW) in Da was calculated in triplicate for each peptide in the non-deuterated sample (ND Control) and at each time point of exchange. The SD is reported. The change in peak width (ΔPW) was calculated by subtracting the PW of the control from the PW of the sample. Bimodality was assessed by taking the maximum peak width for a particular peptide (ΔPWmax) and assessing if it was greater than 2 Da. Peak width was calculated using a method similar to Weis et al. (https://link.springer.com/article/10.1016/j.jasms.2006.05.014). Peptides were centroided with the Apex3D algorithm using DynamX (Waters). Following manual curation, ion stick data were transferred into Excel as two columns of data, m/z values and intensities, and the maximum peak in the isotopic envelope was determined. The list was then searched in descending m/z order to identify the two lowest m/z peaks that straddled 20% of the maximum peak intensity. The m/z value at an envelope intensity of 20% of the maximum intensity was determined using linear interpolation between these two peaks. This process was repeated with a search in ascending m/z order. The relative peak width was determined by multiplying by z (the charge state). For peptide spectra without peaks straddling 20% of the maximum peak intensity on one side of the maximum, typical for lower m/z peaks for peptides exhibiting low deuteration, the farthest isotopic centroid peak on that side was used as the m/z limit for calculating peak width while the other m/z limit was determined using the previously described method.

| **Peptide** | | | | **ND Control** | | **10 s HDX** | | | **10^2^ s HDX** | | | **10^3^ s HDX** | | | **10^4^ s HDX** | | | **Possible Bimodality** | |
| --- | --- | --- | --- | --- | --- | --- | --- | --- | --- | --- | --- | --- | --- | --- | --- | --- | --- | --- | --- |
| **Start** | **End** | **Sequence** | **Length** | **PW**  **(Da)** | ***SD*** | **PW**  **(Da)** | ***SD*** | **ΔPW**  **(Da)** | **PW**  **(Da)** | ***SD*** | **ΔPW**  **(Da)** | **PW**  **(Da)** | ***SD*** | **ΔPW**  **(Da)** | **PW**  **(Da)** | ***SD*** | **ΔPW**  **(Da)** | **ΔPW_max_**  **(Da)** | **ΔPW_max_**  **> 2 Da** |
| 291 | 305 | CALDPLSETKCTLKS | 15 | 2.8 | *0.1* | 4.7 | *0.2* | **1.9** | 4.7 | *0.0* | **1.9** | 5.4 | *0.1* | **2.6** | 5.5 | *0.3* | **2.7** | **2.7** | ✓ |
| 553 | 565 | TESNKKFLPFQQF | 13 | 2.8 | *0.0* | 7.0 | *0.1* | **4.1** | 7.0 | *0.2* | **4.1** | 6.2 | *0.3* | **3.3** | 6.2 | *0.4* | **3.4** | **4.1** | ✓ |
| 553 | 568 | TESNKKFLPFQQFGRD | 16 | 3.5 | *0.1* | 8.5 | *0.1* | **5.1** | 8.7 | *0.1* | **5.2** | 7.3 | *0.2* | **3.8** | 7.2 | *0.1* | **3.7** | **5.2** | ✓ |
| 634 | 642 | RVYSTGSNV | 9 | 1.8 | *0.1* | 5.9 | *0.3* | **4.1** | 5.9 | *0.1* | **4.1** | 5.5 | *0.2* | **3.7** | 5.8 | *0.2* | **4.0** | **4.1** | ✓ |
| 634 | 643 | RVYSTGSNVF | 10 | 1.9 | *0.1* | 6.1 | *0.0* | **4.2** | 5.9 | *0.1* | **4.0** | 6.1 | *0.1* | **4.2** | 5.7 | *0.4* | **3.8** | **4.2** | ✓ |
| 668 | 692 | AGICASYQTQTNSPGSASSVASQSI | 25 | 3.4 | *0.1* | 7.4 | *0.3* | **3.9** | 8.1 | *0.4* | **4.7** | 7.9 | *0.1* | **4.5** | 8.0 | *0.2* | **4.6** | **4.7** | ✓ |
| 870 | 878 | IAQYTSALL | 9 | 1.8 | *0.0* | 2.6 | *0.2* | **0.9** | 2.7 | *0.2* | **1.0** | 2.9 | *0.1* | **1.1** | 2.8 | *0.1* | **1.0** | **1.1** | 🗶 |
| 902 | 916 | MAYRFNGIGVTQNVL | 15 | 3.1 | *0.2* | 5.8 | *0.1* | **2.6** | 6.4 | *0.3* | **3.3** | 6.9 | *0.1* | **3.8** | 7.6 | *0.3* | **4.4** | **4.4** | ✓ |
| 980 | 990 | ILSRLDPPEAEVQ | 11 | 2.1 | *0.1* | 4.0 | *0.2* | **1.8** | 4.7 | *0.1* | **2.6** | 4.9 | *0.1* | **2.7** | 5.4 | *0.3* | **3.2** | **3.2** | ✓ |
| 980 | 992 | ILSRLDPPEAEVQ | 13 | 2.4 | *0.1* | 4.5 | *0.3* | **2.2** | 6.2 | *0.2* | **3.8** | 6.2 | *0.1* | **3.8** | 6.0 | *0.2* | **3.7** | **3.8** | ✓ |
| 982 | 989 | SRLDPPEA | 8 | 1.7 | *0.1* | 3.0 | *0.1* | **1.3** | 3.9 | *0.1* | **2.2** | 3.7 | *0.1* | **2.0** | 3.9 | *0.0* | **2.2** | **2.2** | ✓ |
| 982 | 992 | SRLDPPEAEVQ | 11 | 2.6 | *0.1* | 4.9 | *0.1* | **2.3** | 5.2 | *0.4* | **2.6** | 5.4 | *0.1* | **2.9** | 5.4 | *0.1* | **2.9** | **2.9** | ✓ |
| 991 | 1001 | VQIDRLITGRL | 11 | 2.7 | *0.1* | 4.3 | *0.4* | **1.5** | 4.2 | *0.2* | **1.4** | 5.1 | *0.4* | **2.3** | 7.3 | *0.1* | **4.5** | **4.5** | ✓ |
| 992 | 1001 | QIDRLITGRL | 10 | 2.2 | *0.2* | 3.5 | *0.4* | **1.3** | 3.3 | *0.3* | **1.1** | 4.0 | *0.2* | **1.8** | 5.9 | *0.4* | **3.7** | **3.7** | ✓ |
| 997 | 1006 | ITGRLQSLQT | 10 | 1.9 | *0.0* | 3.0 | *0.0* | **1.2** | 3.4 | *0.2* | **1.5** | 3.8 | *0.1* | **1.9** | 4.8 | *0.1* | **3.0** | **3.0** | ✓ |
| 1007 | 1015 | YVTQQLIRA | 9 | 2.8 | *0.1* | 3.7 | *0.1* | **0.9** | 3.8 | *0.1* | **1.0** | 4.0 | *0.1* | **1.2** | 4.6 | *0.2* | **1.8** | **1.8** | 🗶 |
| 1013 | 1024 | IRAAEIRASANL | 12 | 2.3 | *0.1* | 3.5 | *0.2* | **1.2** | 3.5 | *0.3* | **1.2** | 4.3 | *0.1* | **2.0** | 5.4 | *0.4* | **3.1** | **3.1** | ✓ |
| 1018 | 1024 | IRASANL | 7 | 1.8 | *0.1* | 2.5 | *0.1* | **0.8** | 2.5 | *0.2* | **0.8** | 2.8 | *0.1* | **1.0** | 3.6 | *0.1* | **1.8** | **1.8** | 🗶 |
| 1183 | 1189 | IDRLNEV | 7 | 1.6 | *0.1* | 3.1 | *0.1* | **1.5** | 3.6 | *0.2* | **1.9** | 3.7 | *0.1* | **2.1** | 3.9 | *0.1* | **2.3** | **2.3** | ✓ |
