# Supplementary material for: Identification of a conserved S2 epitope present on spike proteins from all highly pathogenic coronaviruses
Source: eLife. 2023 Mar 21;12:e83710. doi: 10.7554/eLife.83710 (PMC10030117; doi:10.7554/eLife.83710)
Supplement: Supplementary file 4. [file elife-83710-supp4.docx]

**Supplementary File 4a. Antibody variable region sequences**

| **Antibody** | **Region** | **Amino Acid Sequence** |
| --- | --- | --- |
| 3A3 | V_L_ | DIVMTQSAPSVPVTPGESVSISCRSSKSLLHSNGNTYLYWFLQRPGQSPQLLIYRMSNLASGVPDRFSGSGSGTAFTLRISRVEAEDVGVYYCMQYLEYPLTFGAGTKLELK |
|  | V_H_ | QVQLQQSGAELLKPGASVKLSCTASGFNIKDTYIHWLKQRPEQGLEWIGRIGPAIGNTIYAPKFQGKATITTDTSSNTAYLQLSSLTSEDTAVYYCARYYGSSYYYFDYWGQGTTLTVSS |
| RAY53 | V_L_ | DIVMTQSPLSVPVTPGEPVSISCRSSKSLLHSNGNTYLYWFLQKPGQSPQLLIARMSTLASGVPDRFSGSGSGTAFTLKISRVEAEDVGVYYCMQYLEYPLTFGAGTKLEIK |
|  | V_H_ | QVQLVQSGAVVLKPGASVKLSCKASGFNIKNTYIHWLKQAPGQRLEWIGRIGPAIGNTIYAPKFQGKATITTDTSASTAYLELSSLRSEDTAVYYCARYYGSSYYYLDYWGQGTTVTVSS |
| 3E11 | V_L_ | DIVMTQSTSSLSASLGDRVTISCRASQGINNYLNWYQQKPDGTVKLLIYHTSRLHSGVPSRFSGSGSGTDYSLTISNLEQEDIATYFCQQGDTLPYTFGGGTKLEIK |
|  | V_H_ | EVQLQQSGTVLARPGASVKMSCKASGYSFTNYWVHWVKQRPGQGLEWIGAIYPGNGDTTYNQKFKDKAKLTAVTSTSSAYMDLSSLTSEDSAVYYCTRRANYGSRYDYWGQGTTLTVSS |
